# Overwinter and prespawning movements by a vulnerable freshwater pelagophilic minnow

**DOI:** 10.1038/s41598-025-89500-4

**Published:** 2025-03-27

**Authors:** Desiree M. Moore, Shannon K. Brewer

**Affiliations:** 1 Oklahoma Cooperative Fish and Wildlife Research Unit, Department of Natural Resource Ecology and Management, 007 Agriculture Hall, Oklahoma State University, Stillwater, OK 74078 USA; 2 U.S. Geological Survey, Oklahoma Cooperative Fish and Wildlife Research Unit, Department of Natural Resources Ecology and Management, 007 Agriculture Hall, Oklahoma State University, Stillwater, OK 74078 USA; 3 Present Address: U.S. Fish and Wildlife Service, Ash Meadows Fish Conservation Facility, 8757 Spring Meadows Rd, Amargosa Valley, NV 89020 USA; 4 Present Address: U.S. Geological Survey, Alabama Cooperative Fish and Wildlife Research Unit, 3301 Forestry and Wildlife Sciences Building, Auburn University, Auburn, AL 36849 USA

**Keywords:** Freshwater ecology, Animal migration

## Abstract

The decline of pelagophil minnows is related to river fragmentation across the southern Great Plains landscape. Because we know little about pelagophil movement patterns and timing, we aimed to quantify the movements of the vulnerable Arkansas River shiner (ARS) during the winter (November–March) and prespawning (April–June) seasons. We tagged 4233 ARS using visible implant elastomer, passive integrated transponder, or p-Chip micro-transponder tags in 2018–2020. We sampled to recapture tagged fish weekly during the winter and biweekly during the spring. Tagged fish exhibited a downstream movement bias and movement was weakly related to increasing temperature, discharge, and photoperiod during winter, however most of the variability was explained by a random individual effect. Larger individuals moved greater distances than smaller fish. Upstream movements by a migratory portion of the population appeared to begin around late February based on the presence of fish at previously unoccupied sites. However, the first long-distance (30-km) upstream movement by a tagged fish was documented in late May. We show evidence that some ARS may be resident fish at sites throughout winter and spring of multiple years. To conserve freshwater pelagophil minnows, our results indicate water management strategies improving river connectivity in late winter through the spawning season may benefit spawning by migratory individuals, whereas lateral connectivity might benefit the resident portion of the population. Research efforts under experimental flows could provide insight to improved recovery options.

## Introduction

Understanding the movement patterns of fishes can inform conservation and management strategies. Use of fish locations over multiple spatial and temporal extents can provide important information on critical fish habitat and river connectivity needs and can allow us to predict the effects of human alteration. For example, man-made barriers fragment critical habitat and interrupt colonization by small-bodied fishes^1,2^, and Pennock et al.^3^ demonstrated that fishways can be constructed to facilitate upstream passage of diminutive fishes through these barriers. Understanding fish movement and habitat-use patterns can prevent poor conservation and management decisions and outcomes (see^4^ for an overview). For example, biological assessments that do not consider species movement patterns can underrepresent or omit critical life-history stages (e.g., juveniles or reproducing adults) leading to erroneous conclusions about reproduction or recruitment (e.g., minnows and darters^5^).

Fishes make both short and longer-distance movements for several reasons including spawning^6^, juvenile rearing^7^, feeding^8^, accessing refuge environments (e.g., during floods and droughts) and gaining access to other critical habitats^9^. Many populations of fishes include individuals that disperse from their hatching location and resident individuals that remain near their hatching location (e.g., Pecos bluntnose shiner *Notropis simus pecosensis*^10^; Iberian barbel *Luciobarbus bocagei*^11^). The resident portion of a population varies among species (e.g., 20% Pecos bluntnose shiner residents^10^; 89% Iberian barbel residents^11^), by season^12^, may depend on climate conditions^13^, and is an important consideration for management efforts. Fine-scale movements (e.g., within a stream segment) can affect species interaction and are influenced by microhabitat patches offering optimal survival and fish growth^14,15^. Alternatively, coarse-scale movements (e.g., immigration/emigration, long distance movements over several stream segments or between main channels and tributaries) are important for colonization of unoccupied habitat patches^2,16^ and gene flow^17^, and tend to be shaped by long-term evolutionary processes^4,18^. For example, some leuciscids migrate seasonally to increase their long-term fitness (e.g., roach *Rutilus rutilus*; white bream *Blicca bjoerkna*, common bream *Abramis brama*^12^). Understanding movement patterns can provide insight into how fish respond to both natural (e.g., drought) and human perturbations (e.g., introduced species, road crossings), including those factors related to fragmented habitat^2,4^.

Fragmentation in lotic ecosystems is a major threat to the biodiversity of freshwater fauna. Although some barriers can eliminate dispersal (i.e., large dams), some barriers allow passage during some periods (e.g., high flow conditions) but not others. For example, common carp *Cyprinus carpio* passage rates through a lock and dam spillway gate were negligible until discharge reached almost open-river rates^19^. The responses to these so called “semipermeable” barriers^20^ are much more difficult to predict. Extensive droughts, for example, may prevent migrations by fishes to successful spawning habitats^21^ or the successful recruitment of juvenile fishes^22,23^. Recolonization and recovery may be possible, but in the longer term, repeated drought conditions may also result in species extirpations from some river segments^2^. Although species-specific responses (i.e. recruitment, dispersal) to fragmentation and connectivity are expected^24^, fishes possessing certain traits may be more vulnerable.

Some life-history traits in fishes are especially sensitive to river fragmentations. Pelagophils, members of a fish reproductive guild who release semi-buoyant eggs into the open water that potentially rely on drift dynamics during development^25,26^ are hypothesized to be declining in response to river fragmentation. Although pelagic spawning is common among fishes in ocean and coastal environments, it is restricted to only a few families in inland freshwater systems^27^. Whereas the effects of habitat fragmentation on larger fishes have been relatively well studied (e.g^28,29^). , , relationships between diminutive fishes and fragmentation have been slower to emerge (e.g^3^). The drift compensation theory often applied to pelagophils predicts larval drift is compensated for by upstream movement by adult fish primarily in spring and summer^30^. Fragmentation is thought to prevent spring and summer pelagophil fish dispersal. Bonner^31^ found a higher proportion of larger, sexually mature Arkansas River shiner *Notropis girardi* (hereafter ARS) in upstream portions of the Canadian River throughout the year suggesting upstream movement over time. At broader spatial and temporal extents, dams and associated reservoirs fragment available habitat where pelagophils were historically abundant but are now likely extinct^2,32^. Many movement studies have focused on the spawning season (i.e., April-September^33^) because of the importance of reproduction and prevalence of summer stream drying (e.g^34^). , . However, the non-spawning season is also important because these periods are often dry^35^, and winter is often a harsh period for fishes^36,37^. Moreover, overwinter survival can affect recruitment the following year^36^ and is critical to short-lived species.

Understanding the timing of fish movements is important to developing effective management plans, especially for vulnerable species. Despite the listing status of many pelagophil fishes, the movement timing of many species has often been assumed or inferred based on indirect or limited evidence^26,38^. Previous studies assumed juvenile pelagophils immediately began upstream movements because their swimming ability is well-developed^10,38^. A proportion (82%) of Pecos bluntnose shiner was found to move upstream during their first year^10^, but only coarse movement was evaluated (i.e., monthly, or seasonal movement over distances > 55 km). Ruppel et al.^39^ used monthly occurrences of age groups to indirectly infer that upstream movement by prairie chub *Macrhybopsis australis* was related to refuge habitat rather than just reproduction. However, Steffensmeier et al.^40^ found little evidence of an upstream movement bias in recaptured individuals. It is also unknown if pelagophil movements reflect round-trip migration or a one-way dispersal^39,41^.

ARS is threatened in the United States and has declined across most of its historical range^26,42^. Movement of ARS, especially during the non-spawning season, is unknown. Previous work attempted to evaluate ARS movement during the spawning season (April-September), but recaptures were rare (< 2% of *n* = 1,505 over two years, Wilde^38^), leaving a large knowledge gap about the movement patterns of ARS and related species^38,43^. Therefore, our study objective was to quantify ARS movement during the non-spawning, winter season (November-March) and the prespawning period (April-June) over two years (winter 2018 through spring 2020). We hypothesized that larger individuals would be more likely to move upstream, and movement would be positively related to environmental cues (i.e., photoperiod, temperature, discharge). We expected upstream movement distances to increase from winter to the prespawning period.

## Results

### Fish recaptures

From winter 2018 through spring 2020, we recaptured 93 tagged ARS. ARS tagged in 2018–2019 had lower recapture rates (0.8%; *n* = 21) when compared to the 2019–2020 season (3.5%; *n* = 72). Although 16 (1.0%) visible implant elastomer (VIE) tagged ARS were recaptured, only five (0.8%) passive integrated transponder (PIT) tagged ARS were recaptured in the first field season. The remaining 72 (3.5%) ARS were tagged with micro-transponders and recaptured in the second field season.

**Fig. 1:**
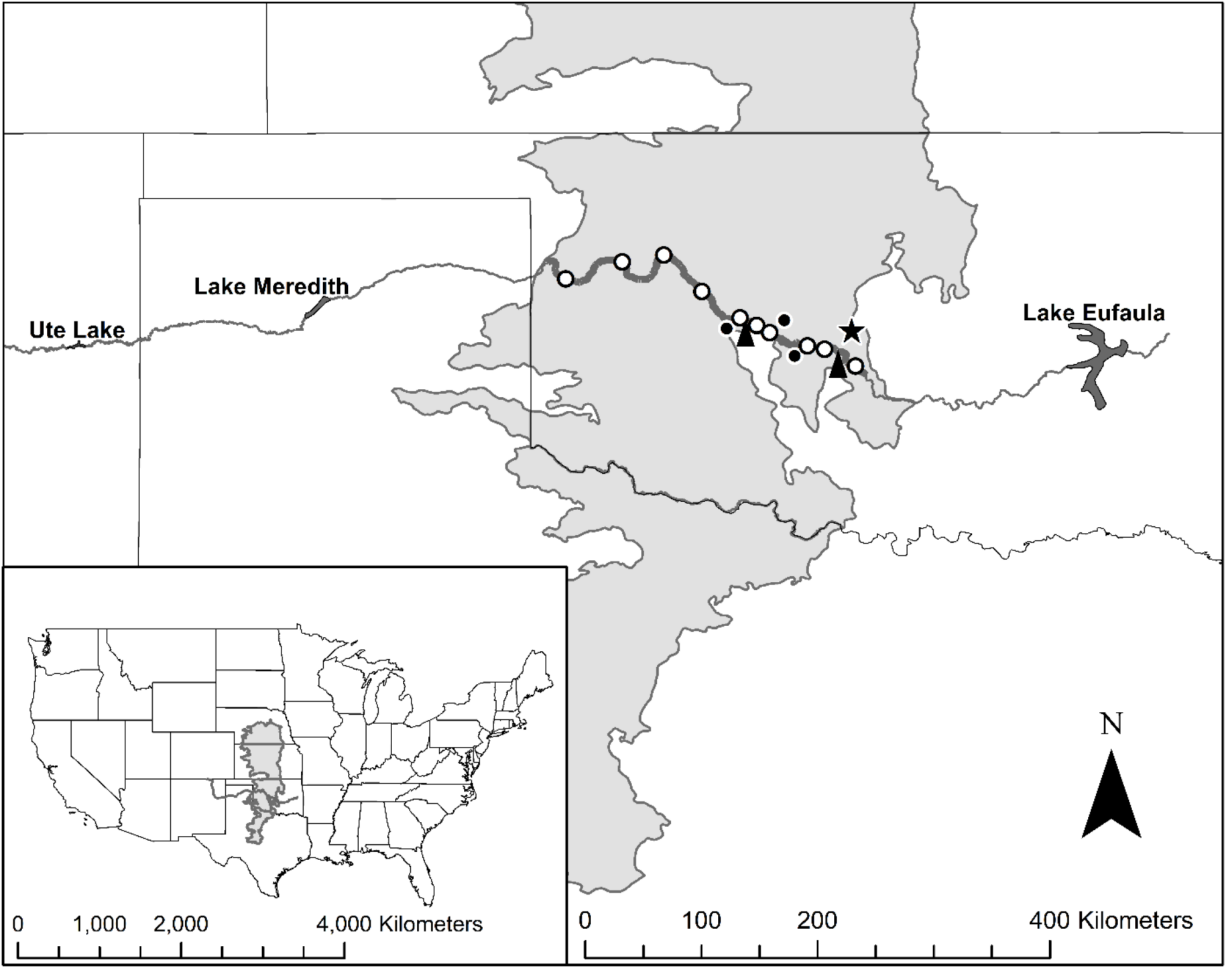
Map of the Canadian River (line) in the Central Great Plains ecoregion (EPA level 3^66^; shaded area). Recapture events occurred between Roll, OK and Purcell, OK (dark gray line). Sites (white dots) from left to right were: (asterisk indicates it is a primary site) Roll, Camargo, Taloga, Thomas, Fire Canyon*, Hinton, Caddo*, Braum’s*, Mustang*, and Norman. Mesonet stations (black dots) are where we retrieved photoperiod data, and U.S. Geological Survey stream gages (black arrows) are where we retrieved discharge data. Oklahoma City is indicated by a star for reference.

Many of our recaptured fish moved short distances; however, some fish that were tagged in the first field season and were recaptured at the same site in the following field season (i.e., a year later). Most recaptured fish (96.8%; *n* = 90) were recaptured within their original tagging site (≤ 885 m; Table 1), some of which were noted to be gravid upon recapture in the spring. Two ARS were recaptured twice at Fire Canyon (see Fig. 1), one and four days after their previous recaptures (40 and 48-mm total length (TL)). There were also three ARS that were VIE tagged during the first field season (November 2018 – April 2019) and recaptured within their tagging site in the second field season (30 October 2019, 8 January 2020, and 4 February 2020), indicating they are either resident fish or exhibit site fidelity during the winter.

**Table 1:** The number of tagged Arkansas River Shiner *Notropis girardi* recaptured within their tagging site (i.e., not including movements between sites) in the Canadian River in the Central Great Plains ecoregion (New Mexico, Texas, Oklahoma) and the direction and average distance (m) moved during each field season (season 1; November 2018-June 2019; season 2; November 2019-June 2020). Winter was defined as November-March, and spring was defined as April-June.

	Season 1		Season 2	
	Winter	Spring	Winter	Spring
Number recaptured	20	1	66	3
Moved downstream	15 (75%)	0	43 (65%)	0
No movement	4 (20%)	0	7 (11%)	0
Moved upstream	1 (5%)	1 (100%)	16 (24%)	3 (100%)
Mean ± SD downstream	− 286.67 ± 168.47	NA	− 192.26 ± 179.64	NA
Mean ± SD upstream	800.00	600	224.53 ± 181.53	430.00 ± 281.54

Average movement distances are reported for upstream and downstream movement (mean ± standard deviation). The percent of fish moving upstream, downstream, or not moving is provided in parentheses.

Some fish tagged in the winter and recaptured in the spring moved appreciable upstream distances (i.e., among sites) during our study. Three ARS recaptured in spring 2020 moved extensive distances over time. An ARS tagged at Mustang on 18 November 2019 was recaptured 91.94 km upstream at Fire Canyon 211 days post tagging. Another ARS was tagged at Fire Canyon on 2 December 2019 and recaptured 49.33 km upstream at Thomas after 190 days. An ARS that was tagged at Caddo on 6 January 2020 was recaptured 136 days later 30.23 km upstream at Fire Canyon. We noted that individuals that moved long distances were gravid upon recapture. Although we could not determine if the long-distance movements were exclusive to the spring season, we did not detect movement between sites in the winter. Additionally, we did not detect long-distance movements in the downstream direction.

### Movement analyses

Individual variability rather than environmental covariates explained most of the variability in mean daily displacement of local movements (within sites) in the winter (November-March). In the top ranked model, ARS had a very weak (R^2^_m_ = 0.0059) negative relationship with temperature, where they moved downstream more with warmer water temperatures (Table 2). Mean daily displacement ranged from − 210 to 205 m (mean = − 15.47 m; SD = 59.27; *n* = 30). Fish also moved downstream more frequently with higher discharge and longer photoperiod (Table 3). Larger fish moved downstream more frequently than smaller fish (− 0.0915; Table 3). Water temperature (0.153) and total length (0.139) had the highest and lowest relative variable importance (RVI) values respectively, but the contributions from all our predictor variables were weak (Table 3). The random individual fish effect explained most of the variability in mean daily displacement (R^2^_c_ = 0.81) indicating that individual variability in movement was high.

**Table 2:** Ranks of candidate linear mixed models evaluating mean daily displacement related to fish total length and environmental variables.

Rank	Model	K	AICc	ΔAICc	Log likelihood	w_i_	$R_m^2$	$R_c^2$
1	Y_ijk_ = β_0_ + γ_*j*_ + β3Temperature_ik_	3	345.04	0.00	− 169.1	0.153	0.0059	0.8102
2	Y_ijk_ = β_0_ + γ_*j*_ + β_4_Photoperiod_ik_	2	345.12	0.08	− 169.1	0.147	0.0001	0.8049
3	Y_ijk_ = β_0_ + γ_*j*_ + β_2_Discharge_ik_	3	345.13	0.09	− 169.1	0.146	0.0063	0.8283
4	Y_ijk_ = β_0_ + γ_*j*_ + β_1_TL_j_	3	345.22	0.18	− 169.1	0.139	0.0009	0.8056
5	Y_ijk_ = β_0_ + γ_*j*_	1	345.32	0.28	− 170.4	0.133	0.0000	0.8251
6	Y_ijk_ = β_0_ + γ_*j*_ + β_2_Discharge_ik_ + β3Temperature_ik_	3	347.63	2.59	− 169.0	0.042	0.0020	0.8167
7	Y_ijk_ = β_0_ + γ_*j*_ + β3Temperature_ik_ + β_4_Photoperiod_ik_	3	347.71	2.67	− 169.1	0.040	0.0038	0.8087
8	Y_ijk_ = β_0_ + γ_*j*_ + β_1_TL_j_ + β3Temperature_ik_	3	347.71	2.67	− 169.1	0.040	0.0062	0.8103
9	Y_ijk_ = β_0_ + γ_*j*_ + β_1_TL_j_ + β_4_Photoperiod_ik_	3	347.75	2.71	− 169.1	0.039	0.0023	0.8052
10	Y_ijk_ = β_0_ + γ_*j*_ + β_2_Discharge_ik_ + β_4_Photoperiod_ik_	3	347.76	2.72	− 169.1	0.039	0.0018	0.8162
11	Y_ijk_ = β_0_ + γ_*j*_ + β_1_TL_j_ + β_2_Discharge_ik_	3	347.80	2.76	− 169.1	0.039	0.0040	0.8230
12	Y_ijk_ = β_0_ + γ_*j*_ + β_1_TL_j_ + β_2_Discharge_ik_ + β_4_Photoperiod_ik_	4	350.34	5.30	− 168.9	0.011	0.0143	0.8407
13	Y_ijk_ = β_0_ + γ_*j*_ + β_2_Discharge_ik_ + β3Temperature_ik_ + β_4_Photoperiod_ik_	4	350.40	5.36	− 169.0	0.010	0.0120	0.8417
14	Y_ijk_ = β_0_ + γ_*j*_ + β_1_TL_j_ + β3Temperature_ik_ + β_4_Photoperiod_ik_	4	350.55	5.51	− 169.0	0.010	0.0059	0.8090
15	Y_ijk_ = β_0_ + γ_*j*_ + β_1_TL_j_ + β_2_Discharge_ik_ + β3Temperature_ik_	4	350.61	5.57	− 169.1	0.009	0.0041	0.8170
16	Y_ijk_ = β_0_ + γ_*j*_ + β_1_TL_j_ + β_2_Discharge_ik_ + β3Temperature_ik_ + β_4_Photoperiod_ik_	5	353.49	8.45	− 168.9	0.002	0.0148	0.8426

Movement data from 30 Arkansas River Shiner *Notropis girardi* were used in the modeling process. For each model, Y is displacement at observation i for fish j, β_0_ is the grand intercept, γ is the random fish effect, TL is fish total length (mm), discharge is the 10-day average discharge (m^3^/s) prior to recapture, temperature is the 10-day average water temperature (°C) prior to recapture, and photoperiod is the number of minutes of daylight on the day of recapture. K is the number of model parameters and comprises both fixed (including interactions) and random effects log likelihood and Akaike’s information criterion adjusted for small sample size (AICc) are reported. ΔAICc was calculated as the difference in AICc score between each model and the top model. The Akaike weight (w_i_) represents the relative support for each model. Marginal R^2^ ($\:{R}_{m}^{2}$) represents the amount of variance explained by the fixed effects, whereas the conditional R^2^ ($\:{R}_{c}^{2}$) represents the variance explained by both fixed and random effects.

**Table 3:** Model averaged results for the top 5 models (ΔAICc < 2) used to assess the relationship between movement of Arkansas River Shiner *Notropis girardi* in the Canadian River in the Central Great Plains ecoregion (New Mexico, Texas, Oklahoma) and our environmental variables.

	Unconditional coefficients	Unconditional SE	Conditional coefficients	Conditional SE	Relative importance
Temperature	− 0.5953	1.3718	− 2.7932	1.6400	0.153
Discharge	− 0.6048	1.4399	− 2.9701	1.7770	0.146
Photoperiod	− 0.0045	0.0107	− 0.0220	0.0132	0.147
Total length	− 0.0915	0.2286	− 0.4712	0.2867	0.139

Unconditional and conditional coefficients and standard error (SE) and relative importance values are reported. Models are listed in table 2.

**Fig. 2:**
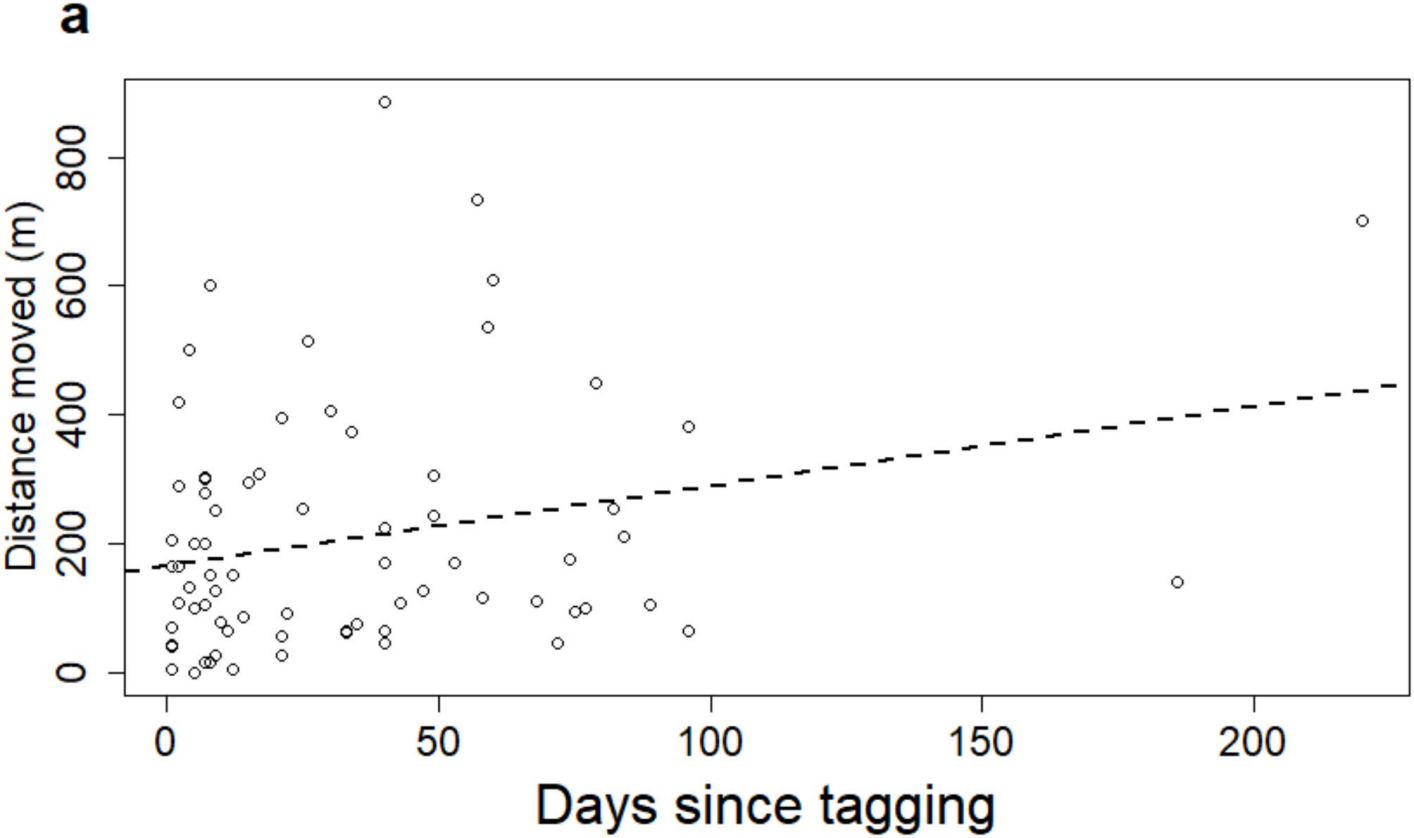
The relationship between days since tagging and non-directional (i.e., absolute value) within-site net distances moved (m) by tagged Arkansas River shiner *Notropis girardi* in the Canadian River, Oklahoma.

We observed temporal trends in net displacement and some net movement patterns related to fish size. We observed that net movement distances, regardless of direction, increased by an average of 1.2 m per day since tagging (P = < 0.01; Fig. 2). However, net movement distances were not related to fish size (*P* = 0.91). The average TL of fish moving upstream (47.3 ± 5.0 mm, range: 40–50 mm) was similar to those moving downstream (46.6 ± 3.7 mm, range: 39–53 mm) (T_23_ = 1.71, *P* = 0.46). However, ARS displaying no movement were smaller, on average, than those that moved (42.7 ± 5.1 mm, range: 36–49 mm) (T_6_ = 1.94, *P* = 0.04).

**Fig. 3:**
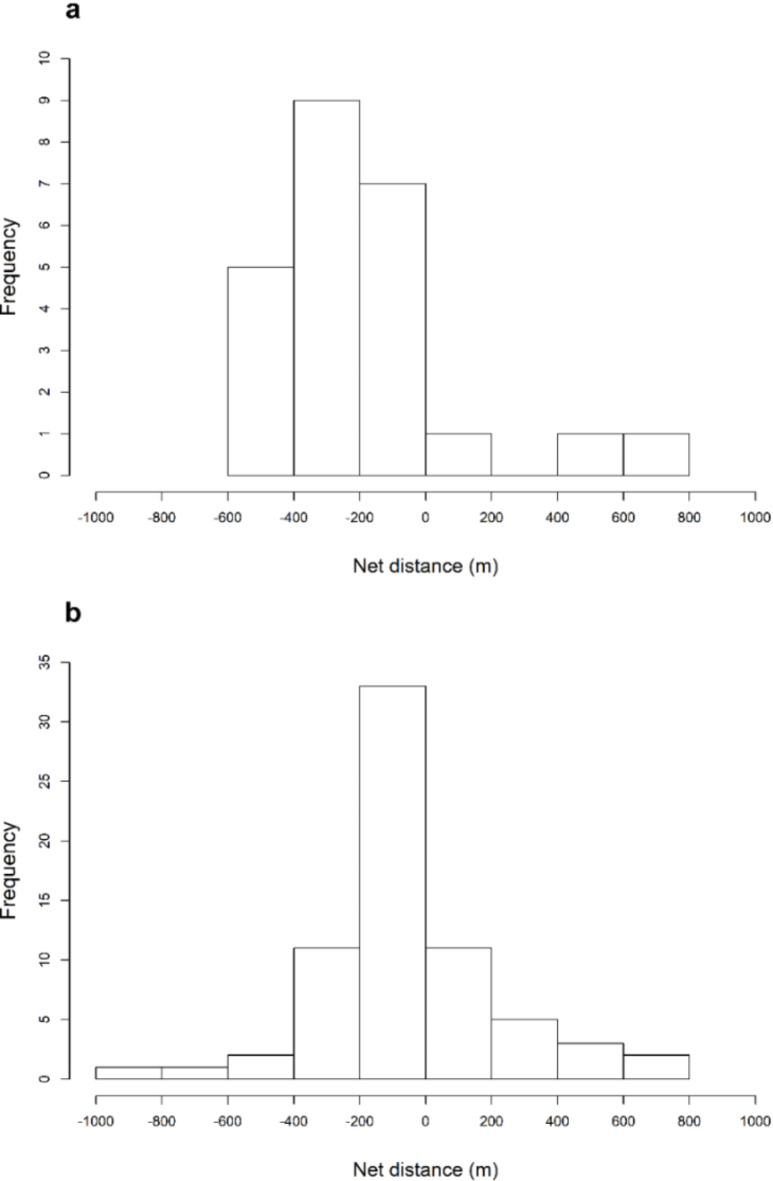
Net distances moved by Arkansas River shiner *Notropis girardi* recaptured within their tagging site (i.e., movements among sites are not included) in the Canadian River in the Central Great Plains ecoregion (New Mexico, Texas, Oklahoma) in (**a**) the first field season (November 2018-June 2019; tagged with PIT and VIE tags) and (**b**) the second field season (November 2019-June 2020; tagged with microtransponders). Negative values indicate downstream movement, positive values indicate upstream movement, and zero indicates the individual was recaptured in the same general area (i.e., 100-m section for season 1 and within 20 m of tagging in season 2) of the river where it was tagged.

We found net movements by ARS during the winter did not display a normal distribution. Results of the D’Agostino Normality Test indicated net distances were not normally distributed (D = 148.18, *P* < 0.01). Instead, the net movement distribution by ARS in both winter and spring was leptokurtic (kurtosis = 65.43, *P* < 0.01). Significantly more ARS (*n* = 58; χ^2^ = 9.29, *P* = 0.002) moved downstream during the winter when compared to upstream (*n* = 17; Table 1; Fig. 3). During the spring, the few tagged ARS we recaptured moved upstream (*n* = 4; Table 1).

Our collection numbers over time indicated there could have been some long-distance upstream ARS movements. During the second field season, we captured zero ARS at Thomas until February 17, but numbers increased thereafter. Similarly, ARS were not captured at Taloga until March 3 when one individual was captured, and numbers slowly increased at that site thereafter. Moreover, we did not catch any ARS at either of the two sites upstream (i.e., Roll and Camargo, Fig. 1) of Thomas and Taloga in either year indicating fish were not moving downstream from these locations. Collectively, these data support that some upstream movements were initiated in February-March.

## Discussion

There has been much debate over the drift compensation theory for pelagophil movements^30,39^. This theory predicts upstream movement by adult fish to compensate for larval drift downstream. However, we found more tagged ARS moving downstream within sites during the non-spawning season. Admittedly, our study design resulted in increased likelihood of capturing finer scale movements (within a site). Previous studies on ARS movement only recaptured individuals over the spawning season (i.e., spring and summer^38,43^), leaving movement patterns over the non-spawning season unknown. Although this study confirmed long-distance upstream movements (30–92 km) by some individuals, these individuals were recaptured in May and June, after the presumed start of the spawning season. Upstream movements also might occur much earlier (i.e., February and March) based on repeated sampling at upstream sites. Moreover, both resident and migratory individuals appeared to be captured in breeding condition. Specifically, we recaptured a tagged gravid ARS during May 2020 at the same site where it was tagged five months earlier (i.e., January 2020) and a gravid ARS tagged in December 2019 was recaptured in June 2020 49.33 km upstream of its tagging location. Although we cannot say if the fish moved between the recapture events, it is more likely that these fish were residents of their capture locations.

Although pelagophils are associated with upstream movement, it appears that ARS have a downstream movement bias during the winter when ARS were only recaptured within their tagging sites. Winter is harsh for fishes due to extreme abiotic conditions and low food availability^36,37^. Fish energy expenditure is typically lower in the winter^44^. Expending the energy to move, particularly upstream, during this time may result in decreased fitness through overwinter mortality^37,45^ or decreased growth and reproductive potential^45^. Other highly mobile fishes also decrease movement in the winter to conserve energy (e.g., bonefish *Albula vulpes*^44^; lake sturgeon *Acipenser fulvescens*^46^). Fishes often resume moving long distances during spring^47,48^. However, in contrast to speculation on pelagophils (e.g., Pecos bluntnose shiner^10^), our results do not support sustained upstream movement immediately following larval development. Platania et al.^49^ found that another pelagic minnow (Rio Grande silvery minnow, *Hybognathus amarus*) also made both upstream and downstream movements in the Rio Grande in New Mexico. Additionally, a downstream movement bias was found for Rio Grande silvery minnow^50^. This finding is an important reminder that movement by a species can vary by season and scale and should be investigated throughout the seasons.

Our results are consistent with the expectation that some unknown portion (*n* = 3 tagged fish in our study) of pelagophils make long-distance (between site) upstream movements during the spring. The long-distance movements detected for ARS were similar to the few previous reports for the species (i.e., 51.7 km^43^; 13.3–213.6 km^38^). The relevant recaptures for ARS occurred in May and June, after the expected onset of the presumed spawning period for these species (i.e., April^43^). We only documented a few extensive movements, which is not surprising given the large size of river and the diminutive nature of the species. It is likely that a higher portion of the population made long-distance movements than we were able to detect. It is noteworthy that we recaptured these fish later than we would have expected given their presumed spawning season; though, limited information is available on inter-annual differences in spawning periodicity as this could vary during wet and dry seasons (e.g., prairie chub^32^). Ruppel et al.^39^ also found movements (inferred by sampling) were inconsistently associated with reproductive effort. Temporal variation both within and among spawning seasons may be an important consideration when predicting population trends. Moreover, we did not capture any ARS at upstream sites until later in the field season and such captures occurred in increasing numbers at two of those sites over time. We also never captured ARS at the most upstream sites indicating these fish were not simply moving downstream. Although, this could be a result of detection bias or some unknown relationship, this is similar to findings of Bonner^31^ and Ruppel et al.^39^ who noted increases in abundances of pelagophils over time. It may be that upstream dispersal is necessary for recolonization, but we found no evidence that a large proportion of the population is making long-distance movements during the winter or the presumed spawning period.

Although we did document long-distance movements by a small proportion of tagged fish, we also found evidence that some ARS may be non-migratory (i.e., residents). Three ARS were recaptured at their tagging site after long periods (i.e., over a year). Although it is possible fish moved among sites between the recapture events, it is more likely that these fish were residents at their capture locations as our chances of detecting a fish returning to a site would be very low. There are life-history tradeoffs between migration and residency^51,52^. Migration may increase recruitment success because propagules require some longitudinal distance to develop while drifting^19,22,53^; however, residents do not expend energy on migration, possibly resulting in better body condition, higher fecundity, and the capacity to spawn on more occasions over a single spawning season^52,54^. The overall effect might be a bet-hedging strategy that heightens overall recruitment^55^. For example, migratory individuals may require greater river fragment lengths to persist over long time frames, but resident individuals may persist in relatively shorter fragments when longitudinal connectivity is decreased, but the eggs and larvae are supported by local complex hydraulic conditions, backwaters, and possibly floodplain access (refer to^26^). Although the proportion of resident fish could not be determined from this study, this is an important area for future research. In particular, it would be advantageous to determine survival associated with the two strategies under different environmental conditions (e.g., high versus low flows). The outcome could lead to more fruitful management strategies for a species occupying a semi-arid region. For example, the importance of lateral floodplain connectivity has been suggested by a few authors^10,53^, but little effort has been devoted to increasing these connections as a management strategy. Given the unlikely removal of dams in this region, exploring alternative strategies for conserving these species seems prudent. Additionally, this is a strategy that could benefit other fishes negatively affected by river fragmentation and loss of longitudinal and lateral connectivity across the world^56^.

The variables we included in the mean daily displacement models were only weakly associated with winter movement. Discharge, temperature, photoperiod, and fish size were all marginally related to daily movement, but random variation among individuals accounted for most of the variation in these data. This is not really that surprising given the variability in these factors during the winter season and the scale over which most movements were made. Moreover, it is not uncommon in fish movement studies for individual variability to be high (e.g^15,57^). , . We hypothesize that as movements become longer, they are more likely to be related to environmental attributes, whereas movements within a site are more likely to be related to food availability, predator presence, and patches useful for improving bioenergetics (i.e., flow refugia)^58,59^. However, we do not have a high enough sample size of long-distance movers in this study to examine this possibility.

Like other studies on small fishes, movements by ARS are potentially underestimated in our study because sample size was limited by the ability to recapture individuals, especially at downstream locations. Fish may have moved upstream and downstream multiple times between recapture events, causing mean daily movement estimates to be lower than the actual values. Because sites are several km apart, individuals that moved shorter distances are easier to capture than individuals that move out of their tagging sites^60,61^. These estimates only use the values of recaptured individuals, causing long-distance movements to be underrepresented in these data. It is common for movement to be underestimated in mark-recapture studies using repeat sampling at relatively few sites^60^. This underestimation was most likely exacerbated in the first field season because the tag types either resulted in higher tag loss or mortality. Moreover, we were unable to sample at the sites furthest downstream sites on many occasions due to flooding. Consequently, it is unclear if the species makes extensive downstream movements. Challenges are difficult to overcome because many pelagic minnows have such small body sizes and the limitations of this study should be recognized when considering the findings.

Micro-transponders may be a suitable field tagging method for small-bodied fishes, especially for those species intolerant of other tag types (e.g., PIT tags). ARS are associated with higher mortality when tagged with PIT tags^62^. Some of this mortality was associated with handling stress, which may have been exacerbated by field conditions (e.g., low temperatures, wind) that were not representative of conditions in a controlled lab setting. The laboratory setting may also provide optimal conditions for recovery, whereas conditions are much harsher in the field (e.g., strong currents, predators, parasites). The recapture rate more than tripled over the second field season when ARS were tagged with micro-transponders, indicating that field mortality and tag loss likely decreased. Micro-transponders can be difficult to use in field conditions because a laptop is currently needed to operate the p-Chip reader; however, successful tagging can be achieved with a tagging station in a safe location such as the riverbank. These results support the broader use of micro-transponder tags in small-bodied aquatic organisms, including young individuals of larger-bodied species, in which other tags have been insufficient.

## Conclusions

Our results indicate that a variety of conservation and management options may be useful for ARS or other pelagic minnow recovery. Our findings indicate that management strategies focused on improving river connectivity by maintaining instream flows via water releases from existing dams in late winter through the spawning season would benefit individuals making upstream movements and propagule drift. This is counter to the typical focus on improving flows only during the warm weather, baseflow season. Different strategies may also be worth considering that could benefit spawning and recruitment by resident members of the population. For example, access to the floodplain has been hypothesized to produce spawning habitat^47,63^; therefore, restoration allowing floodplain access may benefit spawning by resident fish. Although we have limited data, it seems floodplain restoration locations (in our study near river km (rkm) 348) could be beneficial for this species given their extremely limited remaining distribution and the unlikely possibility of removing large dams to improve longitudinal connectivity^26^. We did not capture fish upstream of rkm 379 until late February and March and previous U.S. Fish and Wildlife Service sampling indicated they rarely capture ARS upstream of rkm 250 (Daniel Fenner, oral communication, 2020). Research examining the effects of these types of changes in river connectivity would benefit restoration locations for other threated and endangered pelagophil fishes. Determining flows that maintain longitudinal and lateral river connectivity could benefit recruitment for both migratory and resident populations^22,47^ but may vary in differing climate conditions^64,65^. This approach could also be beneficial to other species with resident and migratory individuals in the population. Given the global effects of dam fragmentation^56^, our findings could be generalized to other riverine species affected by loss of connectivity especially in semi-arid regions). Our results and those of previous studies on pelagophils may be beneficial when developing experimental flows and may also provide a useful context for future research on riverine species having similar reproductive traits.

## Methods

### Study area

All sampling was conducted in the Canadian River in the Central Great Plains ecoregion (i.e., level-three ecoregion^66^) (Fig. 1). The Canadian River begins in eastern New Mexico, flows east through the Texas Panhandle, and terminates in Lake Eufaula, Oklahoma (Fig. 1). The river is characterized by a relatively wide and shallow sand-bed channel and dynamic abiotic conditions (e.g., flood and drought conditions^67^). The river flows from rural areas comprising mixed-grass prairie through agriculture regions upstream of Oklahoma City; these regions are collectively influenced by water withdrawals and large impoundments. The Canadian River has had a > 75% reduction in annual discharge from both Ute and Sanford dams^42,66^. The most common land uses in the upper Canadian River basin are grazing, farming, and oil and gas extraction, whereas the lower basin is heavily urbanized^67,68^.

We tagged ARS at four primary sites on the Canadian River to quantify their movements; however, we added six additional locations as needed to increase recaptures and continue sampling during periodic floods (Table 4; Fig. 1). Our tagging site selection was based on (1) the presence of ARS from recent (i.e., five years) U.S. Fish and Wildlife Service (USFWS) surveys, (2) recent USFWS collection of adult fish, and (3) landowner permissions. All sites were ≈ 1 km in length and split into 10, 100-m sections that were used to examine movement within a site when individual identification was not available (i.e., batch tagged using Visible Implant Elastomer, VIE, described below in ‘Fish Tagging’).

**Table 4:** Summary of tagged Arkansas River Shiner *Notropis girardi* at each site in the Canadian River in the Central Great Plains ecoregion (New Mexico, Texas, Oklahoma) over the duration of our study (season 1; November 2018-June 2019, season 2; November 2019-June 2020): number of tagged fish (N), average size (Mean and standard deviation), and minimum (min) and maximum (max) total length (TL).

Site	*N*	Mean ± SD	Min TL	Max TL	Lat	Long
Season 1						
Roll^*a*^	0				35.8696	− 99.7277
Camargo^*a*^	0				36.0013	− 99.2908
Taloga^*a*^	57 (29)	49.34 ± 2.78	44	57	36.0540	− 98.9689
Thomas^*a*^	137 (68)	48.58 ± 3.41	31	57	35.7716	− 98.6735
Fire Canyon	514 (166)	47.35 ± 3.87	32	60	35.5675	− 98.3781
Hinton^*a*^	0				35.5101	− 98.2504
Caddo	588 (152)	46.30 ± 4.66	33	63	35.4534	− 98.1492
Braum’s	285 (78)	45.88 ± 4.00	35	56	35.3512	− 97.8567
Mustang	436 (78)	44.51 ± 4.99	30	59	35.3255	− 97.7242
Norman^*a*^	194 (49)	45.95 ± 4.71	32	58	35.1943	− 97.4845
Total	2211(620)	46.36 ± 4.49	30	63		
Season 2						
Fire canyon	528	47.12 ± 3.70	37	58	35.5675	− 98.3781
Caddo	691	45.04 ± 4.34	35	58	35.4534	− 98.1492
Braum’s	642	45.09 ± 4.80	36	59	35.3512	− 97.8567
Mustang	161	45.90 ± 4.41	37	59	35.3255	− 97.7242
Total	2022	45.67 ± 4.43	35	59		

^*a*^secondary sites added to increase tagging and recapture rates.

Sites are listed in upstream to downstream order. All fish from season 1 were tagged with visible implant elastomer and fish ≥ 50 mm total length were also tagged with 8-mm passive integrated transponder (PIT) tags in the peritoneum in parentheses. All fish from season 2 were tagged with micro-transponders. Sites are listed from upstream to downstream (see Fig. 1).

### Tag selection

This study was performed under the auspices of the Institutional Animal Care and Use Committee at Oklahoma State University with the approved animal care and use protocol # AG-18-12. All experimental protocols were approved in advance and were performed in accordance with relevant guidelines and regulations. Our methods are reported in accordance with ARRIVE guidelines.

**Fig. 4:**
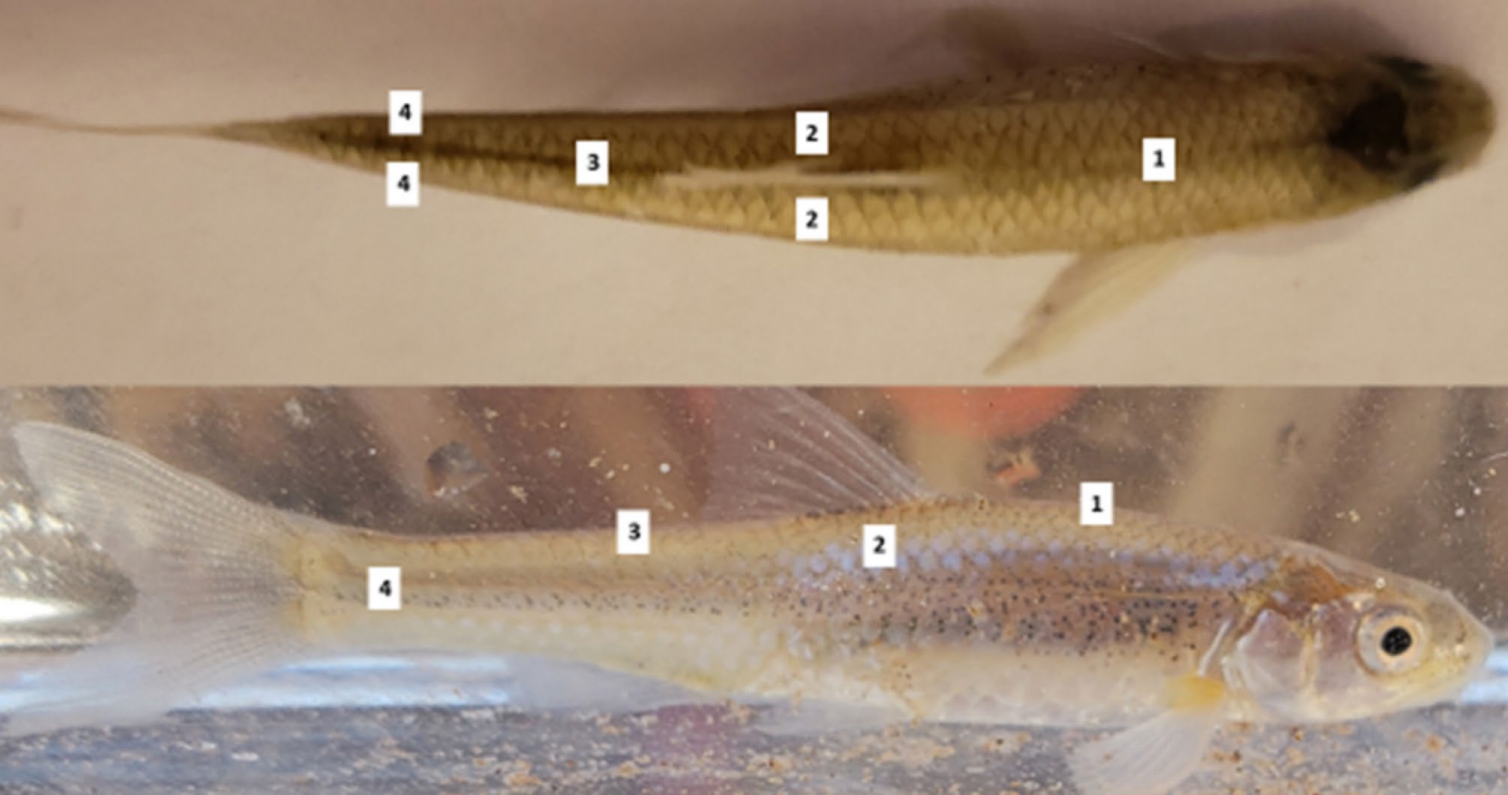
Photographs of an Arkansas River shiner *Notropis girardi* showing the visible implant elastomer (VIE) tag locations. The top panel shows the overhead view, and the bottom panel shows the right side of the fish. Tag locations are (1) nape – anterior to the dorsal fin, (2) dorsal – laterally adjacent to the dorsal fin, (3) rear dorsal – posterior to the dorsal fin, (4) caudal – on the caudal peduncle (photograph credit: Desiree Moore).

We tagged fish in laboratory conditions to determine the best approach to mark fishes^62^. Fluorescent VIE tags allowed tagging of many individuals of a variety of sizes to examine movement trends. After mixing the elastomer, the tag was injected subcutaneously forming a line in two of six possible locations (Fig. 4). We used four different VIE colors: red, yellow, orange, and blue, to represent the four primary tagging sites. We chose these colors because they are the easiest to distinguish from one another once fish are tagged^69^. This tagging scheme allowed us to examine longitudinal movements of 100 m or more when fish moved out of their original 100-m tagging section. ARS that were ≥ 50-mm TL were also PIT tagged based on previous ARS tagging in a lab setting^62^. PIT-tagged individuals were double tagged (i.e., two VIE and one PIT tag) to allow these fish to be used for all analyses. PIT tags are associated with relatively high retention and low mortality in many species, including select small-bodied minnows^70,71^. PIT tags allowed individual identification so we could relate movement to individual size (TL), but this tag could only be used for larger fishes. We determined VIE (Northwest Marine Technology, Shaw Island, Washington) and PIT (Oregon RFID, Portland, Oregon) tags were appropriate for this study based on tagging of a similar-sized minnow; however, we later learned the retention rate in ARS was relatively low (45–50%^62^).

Following low recaptures of marked fishes during the first field season (0.7%), we tested a relatively new, smaller tag (i.e., p-Chips micro-transponders^62^). Because these tags were associated with higher retention (72%) and survival (87%) in ARS and provided individual identification, we tagged fish using micro-transponders during the second field season. Using micro-transponders allowed us to tag many individuals of a variety of sizes and provide individual identification of all recaptured individuals. We could also observe finer-scale movements (≥ 20 m) using GPS (Garmin eTrex Vista C, Lenexa, Kansas) data for tagging and recapture locations (i.e., rather than relying on batch tagged fishes in sections based on tagging location).

### Fish tagging

We used a 3.5 × 1.2-m seine with 3-mm mesh weekly during autumn and winter (November-February) to collect ARS for tagging. The seine was pre-soaked in VidaLife (Western Chemical Inc., Ferndale, WA) to reduce handling stress on fishes (i.e., reduces friction). Sampling began at the downstream end of each site. Seine hauls ≈ 10-m long were completed within a variety of habitats, targeting those where pelagophil species were likely to be found (low-velocity slackwater and deep non-moving pools^26,72^). Seining was conducted using standardized sampling techniques^73^. Collected fishes were held in aerated stream water in a cooler until they were anesthetized for tagging.

**Fig. 5:**
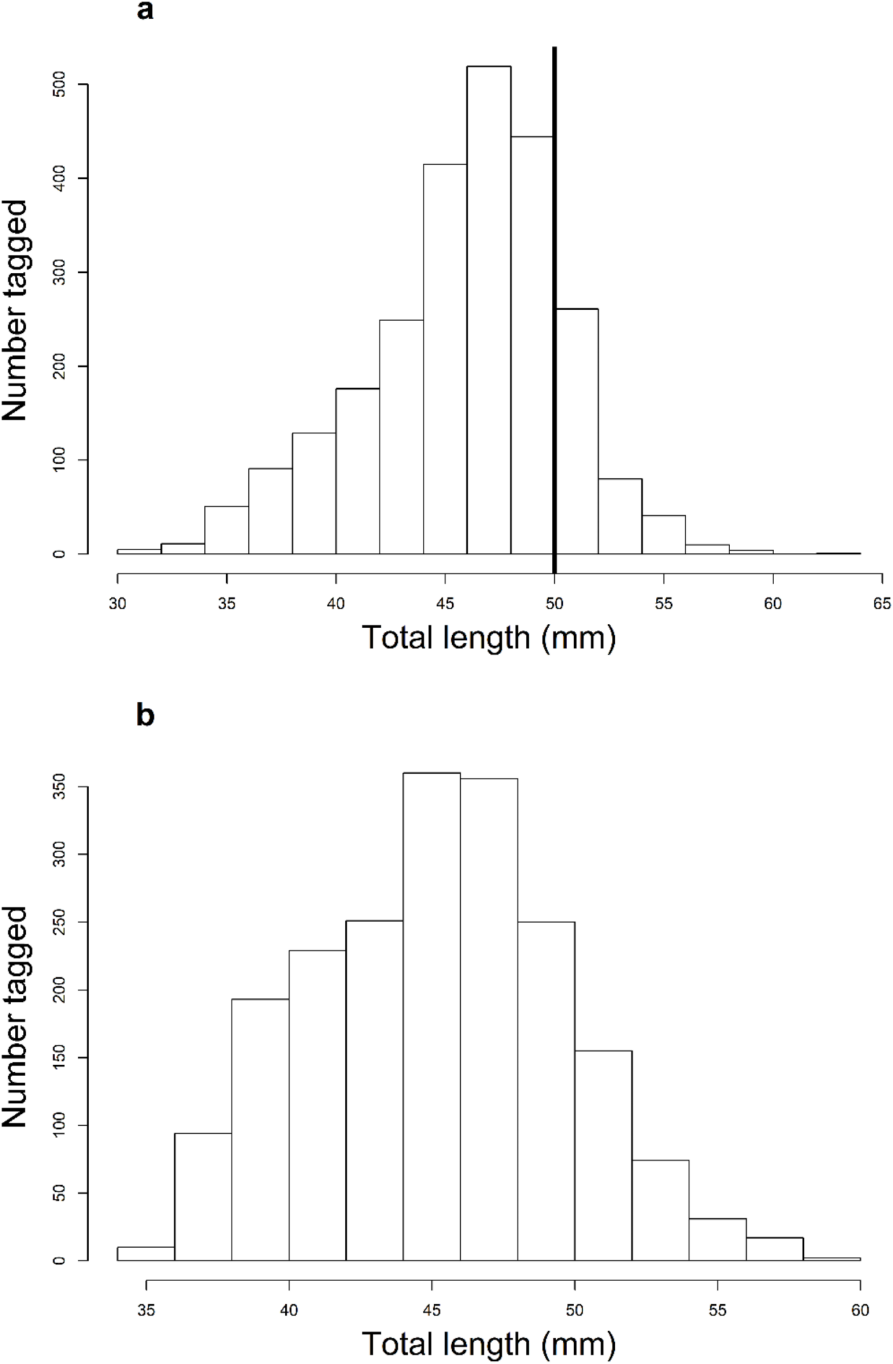
(**a**) The number of Arkansas River shiner *Notropis girardi* tagged in the first field season displayed using total length in 5-mm bins. Left of the black line are individuals tagged with visible implant elastomer (VIE) tags only and right of the line are individuals tagged with both VIE and 8-mm passive integrated transponder tags (PIT) that were inserted into the peritoneum. (**b**) The number of Arkansas River shiner tagged in the second field season (5-mm bins). All individuals were tagged with micro-transponders that were injected subcutaneously.

For the duration of our first field season (November 2018 – April 2019), we tagged 2,211 fish with fluorescent VIE and PIT tags to examine movement. Most ARS captured were 45-50-mm TL (75%), with 25% of ARS large enough to be PIT tagged (Fig. 5). Mean TL of ARS generally increased as we moved upstream (Table 4), and thus higher proportions of larger ARS (≥ 50-mm TL) were tagged at upstream sites. As expected, we captured more ARS at sites where we were able to sample more often (Table 4). We did not capture or tag any ARS at the two most upstream sites (Fig. 1), despite repeated sampling.

During the second field season (November 2019–March 2020), we tagged 2,022 fish with micro-transponders to increase recaptures (Table 4). We captured higher numbers of ARS at the four primary tagging sites in 2019–2020 than in 2018–2019. Thus, tagging was not conducted at the secondary sites during our second field season. Unlike the first field season, mean TL did not show an apparent trend among sites (Table 4). We tagged more ARS at sites we were able to sample more (Table 4).

We anesthetized and tagged fish following Moore and Brewer^62^. Fish were anesthetized using tricaine methanesulfonate (MS-222) at 100 mg/L buffered with sodium bicarbonate to match the pH of the stream water (≈ 200 mg/L). We submerged each fish in the anesthesia solution until it lost equilibrium and operculum movements slowed (i.e., approximately 1–2 min). Then, we recorded its total length (TL; to nearest 1.0 mm) and tagged the fish with VIE, PIT, or micro-transponder tag(s) depending on the field season and fish size (i.e., TL). VIE was injected subcutaneously into each fish according to manufacturer guidelines^69^. Following Musselman et al. (2017), we injected a small (8 × 1.4 mm) full-duplex (FDX) PIT tag (Oregon RFID, Portland, OR, USA) into the peritoneum of each ARS ≥ 50-mm TL using a 1.6-mm diameter injection needle. Over the second field season, we injected each fish with a p-Chip subcutaneously left of the base of the dorsal fin using a 0.8-mm diameter injection needle according to manufacturer guidelines^74^. All tagged fish were immediately placed into an aerated cooler for recovery. Once all normal behavior resumed, fish were released near their capture location (i.e., the sampled habitat in the 100-m section of stream where they were captured). To reduce the stress on fish, water-to-water transfers were made among all holding containers and during release^75,76^.

### Fish recaptures

We seined weekly between November and March to recapture tagged fish to examine both fine (≥ 20 m within sites) and coarse (among sites) movement patterns. More secondary sites were sampled upstream of the primary sites due to extensive downstream flooding. Because fish tagged only with VIE were batch tagged by 100-m section within sites, the resolution for their movements is coarser than when individual identification was possible. Each site was divided into 100-m sections to detect local movements (100 m or more) of batch tagged fish within the sites. The GPS locations of individually identifiable fish (i.e., PIT or micro-transponder tagged) were used to obtain finer movement resolutions (20 m or more). To maintain consistency, we established bin boundaries on the first site visit and returned to these bins throughout the study. We selected and seined transects using the same standardized sampling methods described for tagging^62^. Transects were placed 10-m apart, perpendicular to stream flow within each bin. We randomly selected 8 of the 10 transects within each bin to seine on each sampling event. We conducted seine hauls 10-m apart along each selected transect. Our initial sampling location on each transect was chosen using a random number between 1 and 10 where each number represented the initial sampling location (meters) from the access location. Each seine haul thereafter was 10-m from the first to ensure we were not chasing fish to different locations. We sampled weekly to increase recapture rates as previous research only conducted sampling 1–2 times a month (~ 1% recapture^38^). Although we attempted to sample sites weekly, this was not always possible due to weather conditions, river discharge, or landowner permissions.

Additional sampling was conducted 1–2 times per month April-June to determine the timing of long-distance fish movements (i.e., among sites). We did not anticipate recapturing enough long-distance movements for quantitative analyses. Rather, we wanted to qualitatively assess when long-distance movements were initiated.

We carefully checked recaptured fish for VIE, PIT tags, and micro-transponders while avoiding excess handling stress during identification. We transferred recaptured fish to a prepared cooler of stream water with aeration. Captured fish were removed from the cooler individually to avoid PIT tag interference. Each fish was visually inspected for VIE tags and micro-transponders and scanned with a portable PIT tag reader. If a micro-transponder was located, it was scanned with the handheld laser reader. A shallow dish was used to keep the fish in water during inspection. The identification code of individuals and the site section and GPS location where the fish were recaptured were recorded. We also recorded the breeding condition of all recaptured fish. We considered individuals gravid if oocytes were readily expressed. All fish were released near their approximate capture location (i.e., sample transect) after processing.

### Environmental covariates

We collected water temperature, discharge, and photoperiod data to relate to fish movement. Temperature data were collected at each site using Hobo Pendant MX Water Temperature Data Logger (Onset Computer Corporation, Borne, Massachusetts). Water temperature data (0.01 °C) were recorded hourly and downloaded on each site visit. Discharge data were obtained from the U.S. Geological Survey (USGS) stream gage nearest to each site and converted to m^3^/s (Fig. 1). Sunrise and sunset times were retrieved from the nearest Mesonet station to each site to calculate photoperiod (Fig. 1).

Discharge, water temperature, and photoperiod varied across recapture events from November-March of 2019–2020. Only data for the second field season were used because there were too few recaptures with individual identification in the first field season. The 10-day average discharge ranged from 4.90 to 12.19 m^3^/s (mean = 7.59 m^3^/s; SD = 1.72). The 10-day average water temperature ranged from 4.75 to 10.66 °C (mean = 6.83 °C; SD = 1.69). Photoperiod was right skewed, as expected, ranging 986–1177 min (mean = 999.22 min; SD = 25.50).

### Movement analyses

We calculated environmental variables to relate to ARS movement. Because movement cues may occur several days before tagged fish are recaptured, we averaged our environmental variables over a 10-day period before each recapture event. First, we used hourly temperature and discharge data to calculate average daily temperature and discharge. Then, we used daily temperature and discharge for the 10 days prior to each fish recapture to calculate a 10-day average. We calculated photoperiod as minutes between sunrise and sunset on the day of recapture.

We used movement data to calculate mean daily displacement. Because a fish may move in different directions over time, we only included fish recaptured within 14 days of tagging or previous recapture events to reduce inaccurate calculations of mean daily displacement; thus, all fish recaptured more than 14 days after tagging or previous recapture were excluded. GPS data associated with fish captures were projected in ArcMap (10.2.1, ESRI, Redlands, California) and the longitudinal river distance (10 m) between captures was measured using the Locate Features Along Routes tool. We calculated mean daily displacement as the distance between two consecutive captures, scaled by the number of days between captures^15,77^.

We made appropriate transformations, standardizations, and checked statistical assumptions associated with linear mixed models. Photoperiod was natural-log transformed due to a right-skewed distribution. All variables were standardized to a mean of zero and variance of one to improve model coefficient interpretation. The Pearson’s product moment coefficient |r| was ≤ 0.42 in all pairwise combinations of predictor variables. We confirmed that residuals were normally distributed by examining a histogram of residuals and a qq-plot^78^. We also plotted the residuals against predicted values to confirm the error variance was homoscedastic^78^.

We developed a candidate set of 16 linear mixed models^79^ to assess the relationship between movement (i.e., mean daily displacement) and our environmental variables (Table 2). We only used data collected via micro-transponder tagged fish for these models because of the low number of recaptured PIT-tagged individuals. It was not reasonable to include a tag type in the model due to the few fish recaptured with PIT tags. Our candidate model set included all possible combinations with ≤ 4 parameters of the fixed effects: fish TL (mm), average daily discharge (m^3^/s), photoperiod (min), and average daily temperature (°C). Models with > 4 variables were not included because our sample size was too low (i.e., number of recaptures). A random fish effect was included in all models to account for unexplained variation among individuals, the lack of independence between repeat observations on the same fish, and unequal sample sizes among individuals.

We then assessed the candidate model set to select the top-ranked model relating mean daily displacement to fish TL and environmental variables. We fitted all models using the program R version 3.6.2 (R Core Team 2019) using the “lme4” package^79^ and ranked the models using Akaike’s information criterion adjusted for small sample size (AICc^80,81^) in the “MuMIn” package^82^. We averaged models with AICc differences of less than 2 using the “MuMIn” package to calculate unconditional coefficients, standard errors, and relative variable importance values (RVI). Model averaging results in more robust estimates of fixed effects when models are ranked closely in model selection^83^. The RVI values were calculated by summing the Akaike weight values for all averaged models containing that variable^82^. We also used the “MuMIn” package to calculate R^2^ values. We calculated marginal R^2^_m_ to quantify the amount of variance explained by fixed effects and conditional R^2^_c_ to quantify the amount of variance explained by both fixed and random effects in the candidate models^84^.

We described the net movement by all recaptured fish (November-June) and then statistically determined movement directionality. We calculated net displacement as the distance between the original tagging location and the recapture location. We used the bin midpoints for VIE-tagged fish because they were batch tagged; thus, the resolution for these movements is coarser (≥ 100 m) than for PIT or micro-transponder tagged fish (≥ 20 m). We constructed frequency histograms showing directional movement by tag type (i.e., batch tagged data were visualized separately from individual data) where negative values indicated downstream movement and positive values indicated upstream movement^34^. Additionally, we used a generalized linear model to determine if net movement distances were related to days since tagging and total length. A t-test was used to determine if there were differences in TL between fish that moved upstream versus downstream. We also used a t-test to examine possible differences in TL of fish that moved versus those that did not move. Chi-square tests were used to test for movement directionality by ARS^11,34,85^. These analyses were completed using the program R version 3.6.2 base package^86^. The normality and kurtosis of each distribution were tested using D’Agostino’s test for normality using the “fbasics” package^87^ and Anscombe-Glynn’s test of kurtosis using the "moments” package^88^, respectively^85,89^.

## Data Availability

These data are available as a U.S. Geological Survey data release at 10.5066/P948QCEW^90^.
